# Safety and Efficacy of Oral Direct Factor Xa Inhibitors in Patients With Nephrotic Syndrome: Results From a National Retrospective Study

**DOI:** 10.1016/j.ekir.2025.01.042

**Published:** 2025-02-03

**Authors:** Caroline Arches, Arwa Jalal-Eddine, Dimitri Titeca-Beauport, Myriam Dao, Thierry Lobbedez, Philippe Zaoui, Christophe Masset, Dominique Bertrand, Khalil El Karoui, Henri Brenier, Hamza Sakhi, Bastien Peiffer, Vincent Audard, Nizar Joher

**Affiliations:** 1 Département de Néphrologie et Transplantation, Centre de Référence Maladie Rare Syndrome Néphrotique Idiopathique, Hôpitaux Universitaires Henri Mondor, Assistance Publique-Hôpitaux de Paris AP-HP, Créteil, France; 2 Université Paris Est Créteil, Institut National de Recherche Médicale (INSERM) U88. Institut Mondor de Recherche Biomédicale (IMRB), Créteil, France; 3 Service de Néphrologie, Hôpital Foch, Suresnes, France; 4 Service de Néphrologie, CHU Amiens-Picardie, Amiens, France; 5 Laboratoire MP3CV-EA7517, Université de Picardie Jules Verne, Amiens, France; 6 Service de Néphrologie, CHU Necker-Enfants-Malades, Paris, France; 7 Service de Néphrologie, CHU Caen-Normandie, Caen, France; 8 Association pour la Gestion de la Dialyse et des Usagersporteurs de maladies rénales chroniques Meylan, Université Grenoble Alpes, Grenoble, France; 9 Service de Néphrologie, CHU de Nantes, Nantes, France; 10 Service de Néphrologie, CHU Rouen, Rouen, France; 11 Service de Néphrologie, CHU Tenon, Paris, France; 12 Service de Néphrologie, CHU Rennes, Rennes, France; 13 Département Médico-Universitaire 'Médecine', AP-HP, Hôpitaux Universitaires Henri Mondor, Créteil, France

**Keywords:** bleeding, direct oral anticoagulants, nephrotic syndrome, thrombosis

## Abstract

**Introduction:**

The optimal management of thromboembolism (TE) in patients with nephrotic syndrome (NS) remains challenging. Until now, anticoagulation therapy for NS consisted of vitamin K antagonists (VKAs) or heparin. Data on direct oral anticoagulant (DOAC) use in NS are limited, and their safety and convenience have been well-demonstrated in other indications.

**Methods:**

We conducted a multicenter retrospective study of adult patients with NS treated with therapeutic-dose anticoagulation between 2014 and 2022. We compared the incidences of bleeding and TE events between patients receiving DOAC and those receiving VKAs or heparin (standard-of-care [SOC]). Patients with end-stage kidney disease were excluded.

**Results:**

The overall population consisted of 144 patients (median [interquartile range] age of 54 [38–67] years, 34.7% women) with a median albumin level at 1.5 (1.2–1.8) g/dl and a median urinary protein-to-creatinine ratio of 8.8 (5.5–12.3)g/g. Membranous nephropathy was the main NS etiology (45.8%). No significant differences were observed between the DOAC (*n* = 72) and the SOC (*n* = 72) groups. The anticoagulant strategy was primary prophylaxis in 79.2% of patients taking DOAC and 83.3% of patients with SOC (*P* = 0.67). DOAC use was not associated with an increased rate of TE (4.2% vs. 0%, *P* = 0.25) or bleeding events (6.9% vs. 13.9%, *P* = 0.28) compared with the SOC group. Univariate analysis identified female sex, age > 75 years, and anticoagulant exposure > 90 days as risk factors for bleeding.

**Conclusion:**

This study suggests that DOAC are safer and more effective than conventional anticoagulant strategies for both primary and secondary prophylaxis in patients with NS.

## Graphical abstract

**Figure:**
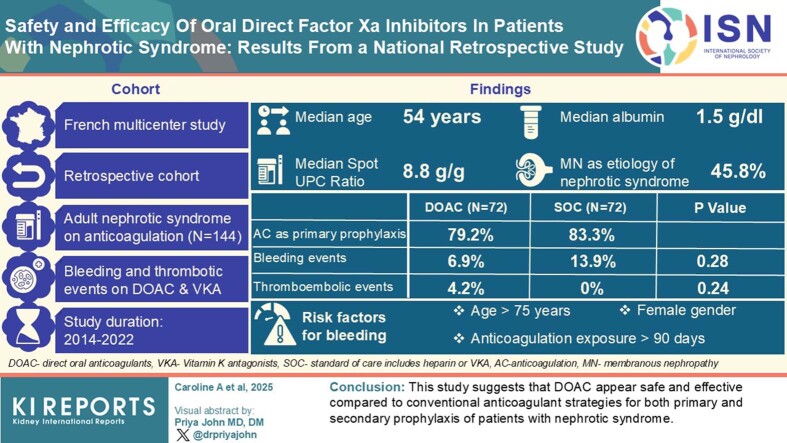

See Commentary on Page 991

TE events are a major life-threatening complication of NS with an overall incidence of approximately 25%.^1^ The pathophysiology of this hypercoagulable state during NS is still incompletely deciphered^2^ but seems to be multifactorial with an imbalance between urinary loss of anticoagulant factors and increased liver synthesis of prothrombotic factors.^1^^,^^3^ NS-associated TE (NS-TE) may be seen in any blood vessel, whether venous or arterial.^4^ However, the most common pattern is observed in the venous system, especially renal vein thrombosis.^1^^,^^5^ The risk for NS-TE varies depending on the underlying glomerulopathy, the severity of hypoalbuminemia, and is especially high within the 6 months following the onset of NS.^6^ Indeed, the risk is particularly high in patients with membranous nephropathy,^7^ serum albumin level < 2 g/dl,^6^^,^^8^ and recent onset of NS.^1^^,^^4^ Although TE is a serious event in the NS course, the optimal prophylactic anticoagulation strategy remains a longstanding conundrum.^6^

Based on international recommendations, when the risk of bleeding is considered to be weak, it is recommended to initiate prophylactic anticoagulation at therapeutic doses in case of serum albumin levels <2–2.5 g/dl using heparin or warfarin.^9^ However, over the past decade, DOACs such as competitive factor Xa inhibitors have emerged as alternative therapeutic approaches to standard anticoagulant strategies.^10^ Meta-analysis of trials, that investigated the efficacy and safety of DOAC in patients with acute TE events, demonstrated that DOACs were noninferior to conventional therapy and associated with a reduced risk of bleeding.^11^ Recently, several international recommendations indicated that DOAC represent the treatment of choice for managing patients with venous TE (VTE).^12^^,^^13^ In addition to the proven safety and efficacy, DOACs have many other potential benefits compared with VKAs, such as a rapid onset of action, a wider therapeutic window, and fewer food and drug interactions.^10^ However, DOACs are cleared by the kidneys and are significantly protein bound in circulation leading to potential modification of their pharmacokinetics in the context of NS.^14^

The existing literature as it stands on DOAC use in NS is limited, including case reports and monocenter or small case series, with DOACs being almost exclusively used for secondary prophylaxis of TE events.^15^, ^16^, ^17^, ^18^ In 2018, Sexton *et al.* published the first case reports describing the use of DOAC for primary prophylaxis against TE in NS.^19^ In this French multicenter retrospective study, including 144 patients with NS, we aimed to assess the safety and efficacy of DOACs as primary or secondary prophylaxis in 72 patients compared with 72 patients receiving either VKA or heparin in the SOC group.

## Methods

### Design and Study Population

We conducted a multicenter retrospective study in 10 French nephrology centers. Inclusion criteria consisted of adult patients with NS defined as hypoalbuminemia < 3 g/dl associated with protein-to-creatinine ratio > 3 g/g on urinary analysis, who were exposed, between 2014 and 2022, to a therapeutic dose of anticoagulant therapy for primary or secondary prophylaxis of thrombotic complications related to NS. We excluded patients undergoing dialysis or with an estimated glomerular filtration rate < 15 ml/min per 1.73 m^2^ using the Chronic Kidney Disease–Epidemiology Collaboration equation at the time of anticoagulation initiation. Patients with long-term exposure to anticoagulants, such as those with atrial fibrillation, were included in the study when they developed NS under this treatment. In such cases, we considered only the events occurring in the NS period and stopped the analysis of medical records when serum albumin levels rose above 2.5 g/dl. Two unmatched groups were established according to the type of anticoagulant treatment as follows: the DOAC group included patients exposed to the oral direct factor Xa inhibitors apixaban or rivaroxaban, and the SOC group included patients receiving VKA or heparin. All patients, irrespective of whether they were undergoing primary (prevention of TE before it occurred) or secondary (to limit progression, embolization, and/or recurrence of TE following diagnosis) prophylaxis, received a therapeutic anticoagulation dose. INR and anti-Xa target ranges were therapeutic ([2–3] and [0.3–0.7 UI/ml], respectively). This multicenter study was performed in accordance with the Declaration of Helsinki and was approved by the Institutional Review Board of Mondor University Hospital (IRB Mondor No 00003835) and the Comité de Protection des Personnesd’Ile-de-France (NO 2016/25NICB).

### Data Collection

Clinical data at the initiation of anticoagulation therapy were retrospectively retrieved from patients’ medical records. Demographic and clinical data included patient age; sex; body mass index; past medical history, particularly previous VTE episodes; type of underlying glomerulopathy; use of antiplatelet drugs; and type and duration of anticoagulant therapy. The HAS-BLED score (Hypertension, Abnormal renal/liver function, Stroke, Bleeding history or predisposition, labile INR, elderly age, and drugs/alcohol concomitantly) was calculated as previously reported.^20^^,^^21^ The HAS-BLED score was used to assess bleeding risk, although it has not been validated in NS. Laboratory parameters included hemoglobin level, platelet count, plasma creatinine level, estimated glomerular filtration rate according to the Chronic Kidney Disease–Epidemiology Collaboration equation, and urinary protein-to-creatinine ratio. All data were recorded on the day of anticoagulant therapy initiation, at any thrombosis or bleeding event, on the day of anticoagulation discontinuation, or at the last available data.

### Outcome

The primary outcome was any reported bleeding events. The secondary outcomes included major bleeding as well as clinically relevant nonmajor bleeding according to the International Society on Thrombosis and Hemostasis criteria,^22^^,^^23^ and TE events. Major bleeding was defined as fatal bleeding, and/or symptomatic bleeding in a critical area or organ, and/or bleeding causing a decrease in hemoglobin level > 2 g/l or leading to transfusion of ≥ 2 units of whole blood or red cells.^22^ Clinically relevant nonmajor bleeding was defined as any sign or symptom of hemorrhage which did not meet major bleeding criteria but at least required medical intervention, hospitalization, increase in care, or prompted a face to face evaluation.^23^ TE events were defined as a new venous or arterial TE event, or a volume increase in preexisting VTE.

### Statistical Analysis

We used frequencies and percentages for categorical variables and medians with interquartile ranges for continuous variables. We used the Mann-Whitney test to compare numeric data and the chi-square or Fisher exact tests for categorical data. Univariate logistic regression analyses were conducted to identify the potential risk factors associated with bleeding. Because of the small number of events, we did not perform a multivariate analysis. All statistical analyses were performed using the R software version 4.3.3 (R Foundation for Statistical Computing, Vienna, Austria). Statistical significance was set at a 2-sided *P*-value < 0.05.

## Results

### Study Population

A total of 144 patients with NS who required anticoagulant therapy were included in this study. Seventy-two patients were administered DOACs, whereas 72 patients were administered VKA or heparin. All baseline characteristics at the time of anticoagulant initiation were comparable between the 2 groups (Table 1). In the DOAC group, the median (interquartile range) age was 58 (38–70) years, and 21 of 72 patients (29.2%) were female. Concerning renal parameters in the DOAC group, we observed a median serum albumin level of 1.6 (1.2–1.8) g/dl, a median urinary protein-to-creatinine ratio of 9.3 (6.1–13.0) g/g, and a median estimated glomerular filtration rate of 75.5 (42.0–99.2) ml/min per 1.73 m^2^. No renal parameters, including NS severity, differed between the 2 groups. Membranous nephropathy was the predominant underlying cause of NS in both groups, accounting for 45.8% of all cases. Detailed treatments forthe underlying glomerulopathy are shown in Table 1.

**Table 1:** Demographic and clinical characteristics before anticoagulation

Characteristics	All (*N* = 144)	DOAC group (*n* = 72)	SOC group (*n* = 72)	*P* value
Age, yr, median (IQR)	54.0 (37.5–67.2)	58.0 (38.0–70.2)	50.5 (35.8–64.2)	0.10
Sex, female, *n* (%)	50 (34.7)	21 (29.2)	29 (40.3)	0.22
BMI, kg/m^2^, median (IQR)	26.8 (23.0–31.0)	26.6 (22.1–30.2)	27.3 (23.6–31.4)	0.35
Active smoking, *n* (%)	22 (17.2)	9 (14.1)	13 (20.3)	0.48
Medical history, *n* (%)				
Hypertension	68 (47.2)	35 (48.6)	33 (45.8)	0.87
Stroke	10 (6.9)	5 (6.9)	5 (6.9)	1
Diabetes	23 (16.0)	9 (12.5)	14 (19.4)	0.36
Hepatopathy	5 (3.5)	1 (1.4)	4 (5.6)	0.21
Heart failure	16 (11.1)	11 (15.3)	5 (6.9)	0.39
Active cancer	10 (6.9)	7 (9.7)	3 (4.2)	0.33
Atrial fibrillation	7 (4.9)	6 (8.3)	1 (1.4)	0.12
Thromboembolic event	14 (9.7)	10 (13.9)	4 (5.6)	0.16
Antiplatelets, *n* (%)	14 (9.7)	8 (11.1)	6 (8.3)	0.78
HAS-BLED score, *n* (%)				0.64
0	83 (57.6)	39 (54.2)	44 (61.1)	
1	43 (29.9)	24 (33.3)	19 (26.4)	
≥ 2	18 (12.5)	9 (12.5)	9 (12.5)	
Hemostasis biological values, median (IQR)				
Platelet count, 10^3^/μl	290 (240–336)	298 (236–343)	282 (245–330)	0.63
PT, %	100 (96–100)	100 (92–100)	100 (100–100)	0.18
aPTT ratio	1.02 (0.95–1.13)	1.02 (0.92–1.12)	1.03 (0.97–1.12)	0.66
Etiology of nephrotic syndrome, *n* (%)				0.49
MN	66 (45.8)	32 (44.4)	34 (47.2)	
MCD	41 (28.5)	20 (27.8)	21 (29.2)	
FSGS	19 (13.2)	9 (12.5)	10 (13.9)	
Lupus	6 (4.2)	2 (2.8)	4 (5.6)	
Amyloidosis	4 (2.8)	4 (5.6)	0 (0.0)	
Other	8 (5.6)	5 (6.9)	3 (4.2)	
Baseline biochemical criteria, median (IQR)				
uPCR, g/g	8.8 (5.5–12.3)	9.3 (6.1–13.0)	8.0 (5.1–11.0)	0.10
Serum albumin, g/dl	1.5 (1.2–1.8)	1.6 (1.3–1.8)	1.5 (1.2–1.9)	0.58
eGFR, ml/min per 1.73m^2^	77.0 (45.0–98.5)	75.5 (42.0–99.2)	78.0 (50.5–95)	0.95
Treatment for renal disease, *n* (%)				
Steroids	80 (55.6)	35 (48.6)	45 (62.5)	0.13
Anti-CD20 mAb	80 (55.6)	37 (51.4)	43 (59.7)	0.40
Calcineurin inhibitor	35 (24.3)	16 (22.2)	19 (26.4)	0.70
Mycophenolate	27 (18.8)	13 (18.1)	14 (19.4)	1
Cyclophosphamide	15 (10.4)	5 (6.9)	10 (13.9)	0.28

aPTT, activated partial thromboplastin time; BMI, body mass index; CKD-EPI, Chronic Kidney Disease Epidemiology Collaboration; DOAC, direct oral anticoagulants; eGFR, estimated glomerular filtration rate using the CKD-EPI formula; FSGS, focal and segmental glomerulosclerosis; HAS-BLED score, Hypertension, Abnormal renal/liver function, Stroke, Bleeding history or predisposition, labile INR, elderly age, and drugs/alcohol concomitantly; IQR, interquartile range (Q1–Q3); mAb, monoclonal antibody; MCD, minimal change disease; MN, membranous nephropathy; PT, prothrombin time; SOC, standard-of-care; uPCR, urinary protein- to- creatinine ratio.

Regarding VTE risks, 10 patients (13.9%) in the DOAC group and 4 patients (5.6%) in the SOC group had a previous history of TE events (*P* = 0.16). In addition, active cancer was present in 9.7% and 4.2% of the patients in the DOAC and SOC groups, respectively (*P* = 0.33). Concerning bleeding risk, 14 patients (9.7%) had an antiplatelet agent associated with the anticoagulant therapy and 18 patients (12.5%) had a HAS-BLED score ≥ 2 in the whole cohort, without statistically significant difference between the 2 groups.

### Anticoagulant Therapy

The anticoagulation modalities are shown in Table 2. All patients were treated with therapeutic-dose anticoagulants. In the DOAC group, a greater proportion of patients was treated with apixaban (*n* = 50, 69.4%) than with rivaroxaban (*n* = 22, 30.6%). Among the patients treated with apixaban, 66% (27/41) and 78% (7/9) received a dose of 5 mg 2 times a day for primary and secondary prophylaxis, respectively (*P* = 0.70). Patients in the SOC group received warfarin, fluindione, tinzaparin, or other heparins in 48.6%, 25%, and 26.4% of the cases, respectively. Anticoagulants were used for primary prophylaxis in 79.2% and 83.3% of the DOAC and SOC groups, respectively (*P* = 0.67), whereas NS-TE was identified at the onset of NS in the remaining cases (15/72, [20.8%] in the DOAC group and 12/72 [16.7%] in the SOC group, *P* = 0.67). Most NS-TE at the onset of NS were pulmonary embolisms; 9 of 15 (60%) in the DOAC group and 8 of 12 (66.7%) in the SOC group (*P* = 1). The other thrombus formation sites are summarized in Table 2. The median duration of exposure was 91 (37–255) days in the DOAC group and 73 (26–362) days in the SOC group (*P* = 0.83).

**Table 2:** Anticoagulant treatment

Anticoagulation features	All (*N* = 144)	DOAC group (*n* = 72)	SOC group (*n* = 72)	*P* value
Type of anticoagulant, *n* (%)				
Apixaban	50 (34.7)	50 (69.4)	-	
Rivaroxaban	22 (15.3)	22 (30.6)	-	
Warfarin	35 (24.3)	-	35 (48.6)	
Fluindione	18 (12.5)	-	18 (25.0)	
Tinzaparin	17 (11.8)	-	17 (23.6)	
Enoxaparin	1 (0.7)	-	1 (1.4)	
Calcium heparin	1 (0.7)	-	1 (1.4)	
Anticoagulant treatment indication, *n* (%)				0.67
Primary prophylaxis	117 (81.2)	57 (79.2)	60 (83.3)	
Secondary prophylaxis	27 (18.8)	15 (20.8)	12 (16.7)	
Pulmonary embolism	17 (63.0)	9 (60.0)	8 (66.7)	1
Deep vein thrombosis	4 (14.8)	2 (13.3)	2 (16.7)	1
Renal vein thrombosis	6 (22.2)	2 (13.3)	4 (33.3)	0.36
Cerebral venous thrombosis	2 (7.4)	2 (13.3)	0 (0.0)	0.49
Arterial thrombosis	4 (14.8)	2 (13.3)	2 (16.7)	1
Duration of exposure to anticoagulation while NS, d, median (IQR)	82 (32–298)	91 (37–255)	73 (26–362)	0.83

DOAC, direct oral anticoagulants; IQR, interquartile range (Q1–Q3); NS, nephrotic syndrome; SOC, standard-of-care.

### Bleeding Events

Among 144 patients, 15 (10.4%) experienced bleeding events during anticoagulation exposure. The characteristics of bleeding events are shown in Table 3. The bleeding rate was lower in the DOAC group than in the SOC group; however, the difference was not statistically significant (6.9% vs. 13.9%, *P* = 0.28). The most common pattern of bleeding events in both groups was mucosal bleeding, including epistaxis (*n* = 4), menorrhagia (*n* = 3), rectal bleeding (*n* = 2), and gingival bleeding (*n* = 1). Regarding major bleeding events, 2 were reported in patients receiving apixaban in the DOAC group and 3 were reported in patients receiving warfarin in the SOC group (2.8% vs. 4.2%, *P* = 1). The 2 patients in the DOAC group were aged > 80 years and had a HAS-BLED score ≥ 2; whereas in the SOC group, the 3 patients were aged 29, 47, and 76 years and had a HAS-BLED scores of 0, 1, and 2, respectively. No difference was observed in the clinically relevant nonmajor bleeding rates between the DOAC and SOC groups (1.4% vs. 4.2%, *P* = 0.62). Readmission or prolonged hospitalization was required for 3 patients in the DOAC group and 6 patients in the SOC group (4.2% vs. 8.3%, *P* = 0.49). The median time (interquartile range) to the onset of a bleeding episode after the initiation of anticoagulation therapy was 44 (20–119) days, with comparable values between the DOAC and SOC groups (31 vs. 45, *P* = 0.44). The characteristics of the patients with bleeding events are summarized in Supplementary Table S1.

**Table 3:** Patient outcomes

Type of bleeding events	DOAC group (*n* = 72)	SOC group (*n* = 72)	*P* value
Bleeding events, *n* (%)	5 (6.9)	10 (13.9)	0.28
Minor bleeding, *n* (%)	2 (2.8)	4 (5.6)	0.68
CRNMB, *n* (%)	1 (1.4)	3 (4.2)	0.62
Major bleeding, *n* (%)	2 (2.8)	3 (4.2)	1
Localization of the bleedings^a^			
Mucosal bleeding, *n* (%)	4 (80.0)	6 (60.0)	
Macroscopic hematuria, *n* (%)	1 (20.0)	5 (50.0)	
Other, *n* (%)	0 (0.0)	2 (20.0)	
Thromboembolic event, *n* (%)	3 (4.2)	0 (0.0)	0.24

CRNMB, clinically relevant nonmajor bleeding; DOAC, direct oral anticoagulant; SOC, standard-of-care.

a Three patients in the SOC group experienced bleeding in more than one location.

In the univariate logistic regression analysis (Supplementary Table S2), the factors significantly associated with an increased risk of bleeding were female gender (odds ratio: 3.22 [95% confidence interval: 1.09–10.18], *P* = 0.04), aged > 75 years (odds ratio: 6.67 [95% confidence interval: 1.78–23.57], *P* = 0.003), and exposure to anticoagulant therapy for more than 90 days (odds ratio: 5.21 [95% confidence interval: 1.57–23.69], *P* = 0.01). However, the type of anticoagulant therapy, coprescription of antiplatelet agents, HAS-BLED score, underlying glomerulopathy, and degree of hypoalbuminemia were not associated with bleeding risk. Because of the limited number of bleeding events, multivariate analysis was not performed.

### TE

TE event rates were similar between the DOAC and SOC groups (4.2% vs. 0%, *P* = 0.24) (Table 3). Notably, all 3 TE events occurred in the DOAC group. One of these involved a 53-year-old patient with genetic thrombophilia—a homozygous factor 2 mutation—who experienced multiple arterial and venous thromboses on day 34 of apixaban anticoagulation. The remaining 2 cases involved patients receiving with rivaroxaban. One patient experienced an ischemic stroke on day 174 and was managed without a change in anticoagulant therapy after neurological recommendations. The last patient, who had been diagnosed with deep vein thrombosis on day 14, was switched to apixaban.

## Discussion

The present study suggests that DOAC-based anticoagulation in NS seems to be safe and effective compared to standard strategies, including heparin and/or VKAs.

DOACs are the cornerstone of anticoagulant strategies in patients with atrial fibrillation or VTE. Indeed, DOACs are superior or noninferior to VKAs in reducing thromboembolic risk and are associated with a lower risk of bleeding.^12^^,^^13^^,^^24^ Despite the exclusion of patients with chronic kidney disease from the most pivotal DOAC trials, DOACs are increasingly being used in this population.^14^ Indeed, many factors contribute to the preferential use of DOACs over heparin or VKA. Apixaban and rivaroxaban are not known to be affected by food and have fewer drug-drug interactions.^10^^,^^25^ In addition, these factor Xa inhibitors have a rapid onset of action with predictable dosing leading to the reduction for routine coagulation monitoring.^10^^,^^25^ Furthermore, concerning the economic burden of the anticoagulation field, DOACs appear to be more cost-effective than standard strategies.^26^^,^^27^ However, the use of DOACs in NS may represent a challenging scenario with safety issues due to their binding to protein.^28^^,^^29^ Hypoalbuminemia has been associated with an increased frequency of bleeding events in patients treated with rivaroxaban.^30^ Nevertheless, in our study we observed only 5 bleeding events (6.9%) in the DOAC group versus 10 (13.9%) in the SOC group. Among these bleeding events, only 2 were considered as major bleeding events in the DOAC group in patients who were particularly frail: an 89-year-old man with a HAS-BLED score of 3 and an 81-year-old female with a HAS-BLED score of 2. This apparent but nonsignificant safer profile of DOACs over VKAs was previously reported by Tijani *et al.* in patients with NS treated with DOAC for primary prophylaxis purposes.^18^ Moreover, a recent study showed no significant differences for total or free apixaban concentrations or antifactor Xa activity for NS (*n* = 8) versus SOC participants (*n* = 11).^31^ Another study showed that apixaban levels were within the therapeutic ranges with a normal intraindividual variability of apixaban levels in 24 patients with NS without any bleeding event reported.^17^ Even if the literature on safety profile of DOAC use in NS is limited, our findings are consistent with published results suggesting that DOAC appear to be safer as a primary or secondary prophylaxis anticoagulation than conventional strategies including heparin and VKA.

NS-TE could be a life-threatening complication, highlighting the major clinical interest of preventing them.^2^ Although the risk of NS-TE is congruent in different studies, it remains widely variable because of renal and nonrenal parameters.^4^^,^^21^^,^^32^ Indeed, several NS-TE–associated risk factors have been reported, such as membranous nephropathy, degree of hypoalbuminemia, female sex, obesity and history of VTE.^33^ Based on these parameters, we can consider that our cohort, including 45.8% of membranous nephropathy with a median of albuminemia < 2g/dl is at very high risk of TE. Despite this apparent high risk, we observed only 3 cases (4.2%) of TE events among patients receiving primary prophylaxis during the anticoagulation period in the DOAC group versus none in the SOC group (*P* = 0.24). Furthermore, we did not notice failure of a secondary prophylaxis strategy in 15 of 72 patients with thrombosis at the onset of NS in the DOAC group. Regarding external data on the efficacy profile of DOACs in NS, we found only 1 multicenter and controlled retrospective case series by Tijani *et al.*^18^ In this study, apixaban and rivaroxaban were used as primary prophylactic strategies in 25 patients with NS compared to warfarin, which was also used as primary prophylactic anticoagulation in 19 patients with NS. The authors reported 1 NS-TE event in each group.^18^ Moreover, we identified 2 monocenter and uncontrolled case series assessing DOACs predominantly as primary prophylaxis strategy: Kelddal *et al.* reported no NS-TE event whereas van Meerhaeghe *et al.* reported 1 pulmonary embolism among 21 and 27 patients, respectively.^16^^,^^17^ Only 1 randomized trial was available in which 16 patients with NS and a documented VTE were allocated to receive either rivaroxaban or heparin as a secondary prophylaxis strategy. Nearly all patients achieved the endpoint of thrombus dissolution.^15^ According to this literature, our study confirmed the potential efficacy of DOACs in the treatment of NS-TE. Although our results did not reach statistical significance, it should be noted that 4.2% of DOAC patients versus 0% in the SOC group displayed TE events (*P* = 0.24). Therefore, clinicians should be aware of the potential risk of thrombosis despite the initiation of anticoagulation therapy at a therapeutic dose.

The emerging concept in the anticoagulation field is to move on to personalized medicine to handle interindividual variability and avoid serious thrombosis or bleeding incidents.^10^^,^^34^ No common variant with a large effect size associated with DOAC-related clinical endpoints has been identified in genetic studies.^10^^,^^35^ Being female and older are associated with bleeding in patients treated with DOACs.^36^^,^^37^ In our cohort, being female and aged > 75 years were risk factors associated with bleeding events in univariate analysis. Concerning comorbidities, chronic kidney disease is associated with elevated systemic exposure to DOACs, particularly with estimated glomerular filtration rate < 30 ml/min per 1.73 m^2^.^10^^,^^28^^,^^29^ Real-world use of DOACs in patients with kidney disease is often associated with dose reduction.^14^^,^^34^ However, Harrington *et al.* showed, in a meta-analysis assessing 71,683 patients treated with DOAC or warfarin in atrial fibrillation in 4 randomized controlled trials, that standard-dose DOACs are safer and more effective than warfarin despite kidney impairment down to a creatinine clearance of at least 25 ml/min.^38^ Moreover, lower-dose regimen of DOACs do not significantly lower the incidence of bleeding compared with standard-dose but are associated with a higher incidence of systemic embolism and death.^19^^,^^38^ Based on these data, the reduction of DOAC dose in patient with NS because of concomitant renal injury or as a precautionary measure may contribute to an increased thrombotic risk. To better personalize DOAC dosing, regular monitoring could be helpful in patients with NS because of NS-associated alterations in the pharmacokinetics of anticoagulation agents.^19^ Nevertheless, the convenience of apixaban administered in fixed doses seems to be preserved regardless of the nephrotic situation and its severity.^17^^,^^31^ Based on our cohort in which 69.4% of patients in the DOAC group were treated with apixaban and on previous studies assessing apixaban,^16^, ^17^, ^18^^,^^31^ apixaban could be a safe and efficacious option in the personalized approach to handle anticoagulation in patients with NS while considering its lower reliance on renal clearance and protein binding than other DOACs.^19^

Our study has some limitations. Its retrospective design is associated with selection bias, errors in data collection, and loss of information such as notable concurrent medications or clinically significant events. In addition, we analyzed data only when patients met the NS criteria and not during the remission phase. In addition, we lacked statistical power because of the low number of events, which did not allow us to perform a multivariate analysis. Notably, it was challenging to constitute an SOC group with recent medical records, suggesting that nephrologists are more likely to prescribe DOAC.

To the best of our knowledge, our study represents the largest study to date comparing DOACs to standard therapies, including heparin and VKA, for primary and secondary prophylactic anticoagulation in patients with NS. The DOAC-based strategy seems to have a safe and efficacious profile in NS-associated primary and secondary prophylaxis, although it is more convenient than standard strategies. To incorporate DOACs into future guidelines on anticoagulation in NS, prospective and larger studies are warranted, although DOACs seem to be integrated into the therapeutic armamentarium for nephrologists. Further studies should explore the use of other DOACs such as direct thrombin inhibitors or edoxaban to better reflect the diversity in international management approaches and practices.

## Disclosure

A received consulting fees for his participation in the Advisory Board for AstraZneca, Vifor Pharma Alnylam, and Theravia outside of the submitted work. All the other authors declared no conflicting interests.

## Supplementary Material

Supplementary File (PDF) **Table S1.** Characteristics of the patients with bleeding events during NS anticoagulation therapy. **Table S2.** Univariate logistic regression analysis of the variables associated with bleeding risk.

## Footnotes

Supplementary File (PDF)
**Table S1.** Characteristics of the patients with bleeding events during NS anticoagulation therapy. **Table S2.** Univariate logistic regression analysis of the variables associated with bleeding risk.
