# Supplementary material for: Safety and Efficacy of Oral Direct Factor Xa Inhibitors in Patients With Nephrotic Syndrome: Results From a National Retrospective Study
Source: Kidney Int Rep. 2025 Feb 3;10(4):1188–95. doi: 10.1016/j.ekir.2025.01.042 (PMC12034858; doi:10.1016/j.ekir.2025.01.042)
Supplement: Supplementary File (PDF) — Table S1. Characteristics of the patients with bleeding events during NS anticoagulation therapy. Table S2. Univariate logistic regression analysis of the variables associated with bleeding risk. [file mmc1.pdf]

Supplementary Material

Supplementary Table S1. Characteristics of patients with bleeding events during anticoagulation for nephrotic syndrome.

Supplementary Table S2. Univariate logistic regression analysis of variables associated with the risk of bleeding.

Supplementary Table S1. Characteristics of patients with bleeding events during anticoagulation for nephrotic syndrome.

						At anticoagulation initiation			At bleeding			
Type of bleeding	Anticoagulant	Age	Sex	HAS-BLED	NS diagnosis	uPCR (g/g)	Serum albumin (g/dl)	eGFR (ml/min/1,73m²)	Delay between anticoagulation and bleeding event (d)	uPCR (g/g)	Serum albumin (g/dl)	eGFR (mL/min/1,73m²)
Major	Apixaban	89	M	3	Unknown	18.7	1.2	39	31	12.3	1.2	42
Major	Apixaban	81	F	2	MN	9.6	0.9	42	117	NA	1.8	39
Major	Warfarin	76	F	2	MCD	18.4	0.9	12*	14	8.7	1.2	18
Major	Warfarin	29	F	0	Lupus	3.3	2.6	87	47	0.5	1.9	39
Major	Warfarin	47	M	1	MN	15.4	0.5	36	379	2.0	0.9	30
CRNMB	Rivaroxaban	32	F	0	Lupus	10.2	1.4	97	141	2.9	2.2	48
CRNMB	Fluindione	82	F	1	MN	NA	1.8	94	200	NA	NA	NA
CRNMB	Fluindione	45	F	0	MN	9.2	2.1	117	44	10.2	1.7	111
CRNMB	Warfarin	56	F	0	MN	7.2	1.7	65	24	3.7	1.3	21
Minor	Apixaban	57	M	0	Amyloidosis	17.0	1.5	102	4	17.0	1.5	102
Minor	Apixaban	68	M	1	MN	21.8	1.0	82	8	21.8	1.0	82
Minor	Warfarin	41	F	0	MN	10.1	1.5	111	28	11.8	1.5	98
Minor	Fluindione	46	F	0	MN	4.7	2.0	61	50	NA	NA	NA
Minor	Fluindione	78	M	2	MN	8.8	1.4	78	449	6.7	1.6	46
Minor	Warfarin	62	M	0	MN	7.5	1.9	95	17	4.3	1.8	96

*Acute kidney injury with eGFR rapidly improving to > 15 ml/min/1,73m².

CRNMB, clinically relevant non major bleeding; eGFR, estimated glomerular filtration rate using CKD-EPI formula; F, female; M, male; MCD, minimal change disease; MN, membranous nephropathy; NS, nephrotic syndrome.

Supplementary Table S2. Univariate logistic regression analysis of variables associated with the risk of bleeding.

**Risk factor**	**Number at risk**	**Bleeding n (%)**	**OR (95% CI)**	***P* value**
Anticoagulant type				
VKA or heparin	72	5 (6.9)	reference	-
DOAC	72	10 (13.9)	0.46 (0.14 - 1.38)	0.28
Sex				
Male	94	6 (6.4)	reference	-
Female	50	9 (18.0)	3.22 (1.09 - 10.18)	0.04
Age				
≤ 75 yr	130	10 (8.0)	reference	-
> 75 yr	14	5 (36.0)	6.67 (1.78 - 23.57)	0.003
HAS-BLED score				
0	83	8 (9.6)	reference	-
1	43	3 (7)	0.70 (0.15 - 2.58)	0.62
≥ 2	18	4 (22.2)	2.68 (0.65 - 9.81)	0.15
Antiplatelets				
No	130	13 (10.0)	reference	-
Yes	14	2 (14.3)	1.50 (0.22 - 6.34)	0.62
Serum albumin, g/dl				
> 1	122	10 (8.2)	reference	-
≤ 1	20	4 (20.0)	2.80 (0.70 - 9.53)	0.11
eGFR, ml/min/1.73m^2				
< 30	13	1 (7.7)	reference	-
[30;60[	41	3 (7.3)	0.95 (0.11 – 20.14)	0.10
[60;90[	40	5 (12.5)	1.71 (0.24 – 34.59)	0.64
≥ 90	49	6 (12.2)	1.67 (0.25 - 33.23)	0.65
Membranous nephropathy				
No	78	5 (6.4)	reference	-
Yes	66	10 (15.2)	2.61 (0.87 – 8.77)	0.10
Duration of anticoagulation				
≤ 90 d	76	3 (3.9)	reference	-
> 90 d	68	12 (17.6)	5.21 (1.57 - 23.69)	0.01

OR, odds ratio; CI, confidence interval; DOAC, direct oral anticoagulants; eGFR, estimated glomerular filtration rate using CKD-EPI formula; VKA, vitamin K antagonist.
